# Pesticide exposures in a malarious and predominantly farming area in Central Ghana

**DOI:** 10.5897/AJEST2015.1912

**Published:** 2015-08-30

**Authors:** Kenneth Ayuurebobi Ae-Ngibise, Patrick L. Kinney, Kwaku Poku Asante, Darby Jack, Ellen Abrafi Boamah, Robin Whyatt, Mohammed Mujtaba, Alexander Manu, Seth Owusu-Agyei, Blair J. Wylie

**Affiliations:** 1 Kintampo Health Research Centre, Ghana Health Service, P. O. Box 200, Brong Ahafo Region, Kintampo, Ghana; 2 Mailman School of Public Health, Columbia University, New York, NY, USA; 3 Division of Maternal-Fetal Medicine, Vincent Department of Obstetrics and Gynecology, Massachusetts General Hospital and Harvard Medical School, Boston, MA, USA

**Keywords:** Pesticide, malaria, prevalence, Kintampo, dichlorodiphenyltrichloroethane

## Abstract

In areas where malaria is endemic, pesticides are widely deployed for vector control, which has contributed to reductions in malaria deaths. Pesticide use for agrarian purposes reduces pest populations, thus improving crop production and post-harvest losses. However, adverse health effects have been associated with pesticide exposure, ranging from skin irritation to neurotoxicity and carcinogenicity. Though misuse of these pesticides can lead to widespread potential dangers, the debilitating effects are usually underappreciated in many developing countries. To evaluate the pattern of pesticide usage among rural communities in the Kintampo area of Ghana, a cross-sectional survey was conducted among 1455 heads of households randomly sampled from among 29,073 households in the Kintampo Health and Demographic Surveillance System area of Ghana to estimate the prevalence of pesticide use and indications for use among this rural populace. Seventy-one percent (1040/1455) of household heads reported having used pesticides on either their farms or homes, most commonly for control of weeds (96.4%, 1003/1040) or insects (85.4%, 888/1040). Dichlorodiphenyltrichloroethane (DDT) was used by 22.9% (238/1040) of respondents. The majority of households who reported use of pesticides said women in their households assisted in the spraying efforts (69.3%, 721/1040); of these women, 50.8% (366/721) did so while carrying their babies on their backs. Only 28.9% (301/1040) of the study participants wore protective devices during pesticide applications. Frequent symptoms that were reported after spraying, included cough (32.3%; 336/1040), difficulty in breathing (26.7%; 278/1040) and skin irritation (39.0%; 406/1040). Pesticide use among community members in the Kintampo area of Ghana is common and its potential health impacts warrant further investigation.

## Introduction

In homes, pesticides are commonly used to control insects and rodents (Jurewicz and Hanke, 2008). Pesticides contain known substances that are used to destroy, repel or control organisms such as insects, weeds, and rodents that are identified as pests. Pesticides are also used during farming to increase agricultural output even by individuals running small scale and subsistence farms (Shafer et al., 2005). Insecticides are also widely deployed to prevent not only diseases caused by insect vectors such as malaria, filariasis, and sleeping sickness but also for their biting nuisance. Insecticide use in malaria endemic areas has contributed to a tremendous reduction in the number of lives lost (Cooper and Dobson, 2007). Insecticides for vector control are applied at the community level during spraying campaigns by governments; but are also commonly used by individuals in their homes (Ranson et al., 2011; Mabaso et al., 2004). Vector control programs mostly rely on the use of insecticides that are applied as indoor residual deposits to treat mosquito nets or curtains (Fuseini et al., 2011). Dichlorodiphenyltrichloroethane (DDT) has been a front-line insecticide for mosquito control and continues to be sanctioned by the World Health Organization (WHO) for indoor residual spraying efforts despite its known persistence in the environment (WHO, 2011, Mohloai, 2006). Though it has in the past been reported that, the main malaria vectors, Anopheles gambiae and Anopheles funestus in Ghana are resistant to DDT, pyrethroids and carbamates (Coetzee et al., 2006; Hunt et al., 2011), little is known about pesticide exposure levels in farming populations living in rural areas where malaria is also endemic.

Worldwide, about three million people suffer from acute severe pesticide poisoning and over 20,000 die each year; the majority of these adverse events occur in developing countries (WHO, 2011; Hough, 2013). Unfortunately, in developing countries consumers are misled by false promotion of the benefits of insecticide use by chemical companies and are not educated about potential dangers posed by widespread misuse of these chemicals (Lyndon, 1989). The regulatory control of chemicals in these settings is inadequate. The consequent increased exposure to these chemicals (Soni et al., 2011) may contribute to poor health outcomes such as abnormal neurodevelopment, male infertility, or cancers, (Koureas et al., 2012; PANAP A Factsheet Series, 2014; Gunnell et al., 2007; Horton et al., 2011).

Ghana is one of the endemic malaria areas in Africa where malaria control is a major issue (Biritwum et al., 2000). Agriculture is the most common economic activity in Ghana (Hart, 1973; Aryee, 2001), contributing to over 50 percent of Gross Domestic Product (Breisinger et al., 2008). These major activities require the use of pesticides, however, the prevalence and frequency of use of pesticides is unreported. We aimed to estimate the frequency of pesticide use in a predominantly farming community in Ghana (Owusu-Agyei et al., 2012) with high malaria burden (Owusu-Agyei et al., 2009) in order to inform regulatory authorities tasked with improving strategies to curb pesticide misuse.

## Materials and Methods

### Study area

The study area is located in the Brong Ahafo Region of Ghana and has a resident population of approximately 140,000 which is largely rural. Subsistence farming is the predominant occupation (Owusu-Agyei et al., 2012). There are two government hospitals, two private hospitals, four health centres, one private clinic, 25 Community-based Health Planning Services (CHPS) zones, and two private maternity homes in the study area. There are lots of chemical shops that sell a variety of chemicals and other pesticides for both domestic and farming activities. Malaria remains the leading cause of outpatient visits in all health facilities located in the study area. Other health problems include respiratory infections and diarrhoeal diseases.

### Study design and participant identification

A cross-sectional survey was conducted among heads of households who were sampled randomly from two adjoining local government districts of Ghana, Kintampo North Municipality and Kintampo South District of Ghana, using the Kintampo Health and Demographic Surveillance as a sampling frame. This information was collected as part of the baseline data required for the Ghana Randomized Air Pollution and Health Study (GRAPHS, Trial Registration NCT01335490), which is assessing the effectiveness of improved cook stoves in increasing birth weight and reducing acute lower respiratory tract infections during the first 12 months of age among woman-infant pairs recruited during pregnancy. The data collection for this survey was carried out in December 2011 using structured questionnaires. GRAPHS involved registered members of the Kintampo Health and Demographic Surveillance System (KHDSS). The KHDSS provides longitudinal data of residents in the study area (Owusu-Agyei et al., 2012); vital information of members is regularly update twice per year to monitor demographic change such as births, deaths and migration into and from the area). Each registered member has a permanent identification number and a household address. A simple random selection procedure involving STATA version 12 was used in selecting respondents from the KHDSS database. A total of 1455 heads of households were selected and identified in their households using the address system of KHDSS.

### Data collection

A pretested questionnaire was used to collect data on pesticide use by households including information about the frequency and purpose of use both in the home and on farms. Data were also collected on symptoms following pesticide application. Each questionnaire was administered by a trained fieldworker with each interview lasting approximately 30 min.

### Ethics

Ethical approval was sought from the KHRC Scientific Review Committee as well as the KHRC Institutional Ethics Committee (Federal Wide Assurance number: 00011103). Written consent was obtained from all study participants. These consent forms were signed or thumb printed by the study participants and countersigned by a member of the research team. Participants who provided consent by thumb print were accompanied by witnesses who were present for the consent process and also signed the consent form.

### Statistical analysis

Data were analysed descriptively using STATA version 12 (StataCorp, College Station, TX, USA).

## Results

**Figure 1:**
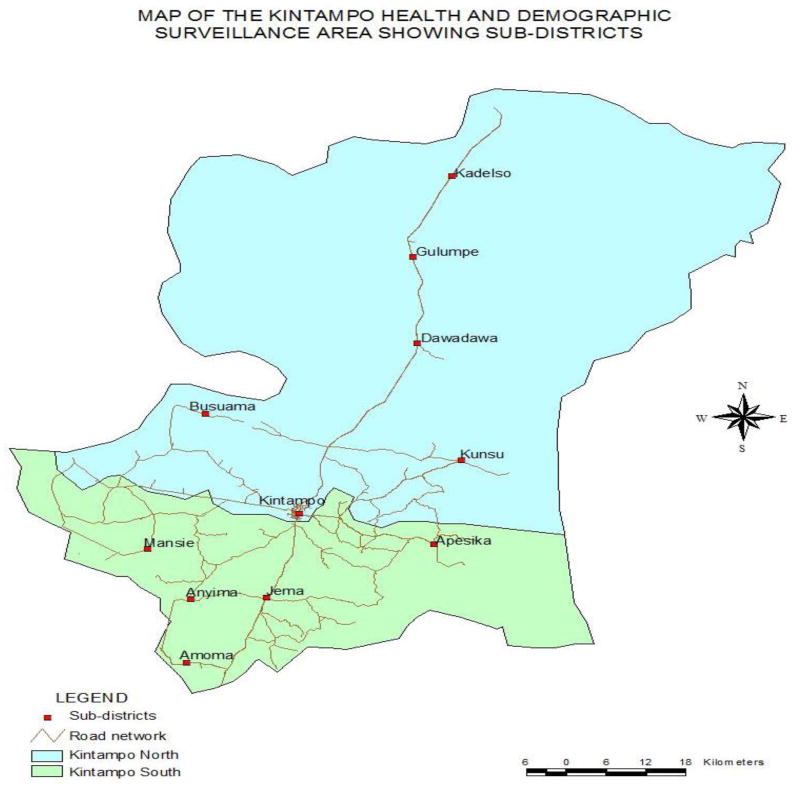
Location of KHDSS showing the Sub-districts, Kintampo, Ghana.

A total of 71.5% (1040/1455) of respondents reported having used pesticides on either their farms or homes for a variety of purposes. The use of pesticides ranged from 30.9 to 88.1%, depending on the sub-districts. It was proportionally highest in the two sub-districts of Dawadawa 88.1% and Anyima 80.5%, and lowest in Kadelso sub-district 30.9% (Table 1). A map of the study area is shown in Figure 1.

The most common class of pesticides deployed were herbicides (96.4%, 1003/1040), followed by insecticides (85.4%, 888/1040) (Table 2). About 84.2% (1225/1455) of the study participants own farms or gardens. Pesticides were frequently applied by study participants with almost one third (29.3%, 305/1040) of households spraying once a month or more frequently (Table 2).

The prevalence of dichlorodiphenyltrichloroethane (DDT) use was not infrequent among respondents (22.9%, 238/1040). Among respondents who used DDT, DDT was used mostly for insect control (90.8%, 216/238), and also used for control of weeds (3.4% of DDT users), rodents (21.0% of DDT users) and head lice (10.5% of DDT users).

The majority of study participants who used pesticides (71.1%, 739/1040) did not wear any protective equipment, such as masks or gloves during the application of pesticides. Most (66.4%) of the pesticides were bought from the open markets. Persons involved in spraying are generally the same people who mix the chemicals for spraying.

Over 69.3% (721/1040) of women assist in spraying of farms and gardens using pesticides. About 50.8% (366/721) of women who assisted in spraying did so carrying their babies on their back (Table 3).

The study participants reported a range of negative health effects after pesticide application (Table 4). The most common symptoms reported were skin irritation (39.0%), headache (37.0), runny nose (36.5%), cough (32.3%) and difficulty in breathing (26.7%).

## Discussion

We found from our survey that the use of pesticides in the Kintampo area of Ghana is quite common. Pesticides are widely available and are applied for various purposes including control of pests and weeds in residents' homes and on their farms by the majority of respondents. It is therefore not surprising that pesticide contamination of food crops in Ghana has been previously reported (Darko and Akoto, 2008). From the lettuce samples analyzed in this study, chlorpyrifos was detected as a residue in 78%, lindane in 31%, endosulfan in 36%, lambdacyhalothrin in 11%, and DDT in 33%.

Unfortunately, our survey also demonstrated that most people using pesticides do not use protective gear during pesticide application and side effects were commonly experienced after application. Women, with their babies attached to their back, often participate in the application of pesticides suggesting even the most vulnerable among the population may be unintentionally exposed. Our findings underscore a need for increased regulatory oversight of commercially available pesticides as well as improved educational programs aimed at pesticide consumers in rural African settings. An emphasis on the use of personal protective equipment during application may alleviate acute side effects and accidental poisonings.

The implications of such widespread pesticide use at the household level on insecticide resistance are unclear. Insecticides remain an integral strategy for malaria control in indoor residual spraying programs and for treatment of bed nets. The burden of malaria is high in the study area with perennial malaria transmission. The prevalence of malaria parasitemia is about 50% among children less than 10 years of age and the entomological inoculation rate was 269 infective bites per person per year in 2009 (Owusu-Agyei et al., 2009). The classes of insecticides used for malaria control-pyrethroids, organophosphates, carbamates-- are similar to those available in chemical shops for individual consumer use. Whether this frequent household use reduces the effectiveness of malaria vector control needs further study.

Another notable finding from our study was how frequent DDT was being used by individuals outside of government malaria vector control programs for purposes other than mosquito control, even for head lice control. The international treaty on persistent organic pollutants was signed on May 31, 2001 in Stockholm by 151 countries, including Ghana; in the Stockholm convention, DDT use is restricted to disease vector control programmes. Our survey highlights that once DDT is available, its application extends beyond control of mosquitoes (Sadasivaiah et al., 2007, WHO, 2011). A study on pesticides contamination among farmers in Ghana found the presence of high organochlorine pesticide residues, including DDT, in the breast milk and in blood samples from vegetable farmers (Ntow et al., 2008). According to this study, some women farmers had accumulated levels of pesticide residues in breast milk above the ‘tolerable daily intake’ guidelines. There is an increased need for policy regulations around availability and application of restricted pesticides like DDT.

### Limitations and strengths of the study

As a descriptive study, our data could not establish causal relationships. We could not demonstrate specific negative effects of exposure to pesticides. This study was not able to clarify the classes of pesticides employed such as organochlorines, organophosphorus, carbamates, and pyrethroids. Nonetheless, there are few studies in Ghana on pesticide use; this study therefore makes a significant contribution to the body of knowledge in this area.

### Conclusion

Pesticide use in the Kintampo area of Ghana is common, and the potential health hazards warrant further study. There is a need to review safety precautions in the use and application of pesticides among rural populations in Ghana. We also recommend further studies to evaluate the association between pesticide exposures pre- and postnatally and neurodevelopment. This information will inform stakeholders to critically examine both the risks and benefits of pesticide use especially in developing regions in the world.

## Figures and Tables

**Table 1:** Prevalence of pesticide use by sub-district in the Kintampo Area of Ghana, December 2011.

Sub-district	Number sampled	Number of pesticides users	Percentage
Dawadawa	42	37	88.1
Anyima	128	103	80.5
Gulumpe	194	155	79.9
Kunsu	60	47	78.3
Jema	283	218	77.0
Kintampo	390	289	74.1
Busuama	113	71	62.8
Mansie	83	50	60.2
Apesika	29	17	58.6
Amoma	65	32	49.2
Kadelso	68	21	30.9
Total	1455	1040	71.5

**Table 2:** Frequency and types of pesticides used by household members in the Kintampo Area of Ghana, December 2011(n=1040).

Parameter	Number	Percentage (%)

Type of pesticides used		
Herbicides	1003	96.4
Insecticides	888	85.4
Rodenticides	648	62.3
**Frequency of use**		
Weekly	26	2.5
Every two weeks	148	14.2
Monthly	131	12.6
Greater than monthly intervals	735	70.7
Use of DDT among households (n=1040)	238	22.9
For the control of insects and pests	216	90.8
For the control of rodents	50	21.0
For the control of head lice	25	10.5
For the control of control weeds	8	3.4

**Table 3:** Practices regarding the acquisition and use of pesticides among households in the Kintampo Area of Ghana, December 2011(n=1040).

Parameter	Number	Percentage (%)

Practices associated with pesticide use		
Use of nose masks, gloves while spraying	301	28.9
Women assist in spraying of farms	721	69.3
Women carry babies on back while spraying	366	50.8
**Places of pesticide purchase**		
Open market	691	66.4
Accredited Vendor	310	29.8
Agricultural extension office	8	0.8
Mobile vendors	31	3.0

**Table 4:** Self-reported symptoms experienced by household members after use of pesticides in the Kintampo Area of Ghana, December 2011(n=1040).

Symptom	Number	Percentage (%)
Skin irritation / rash	406	39.0
Headache	385	37.0
Runny nose	380	36.5
Cough	336	32.3
Difficulty in breathing	278	26.7
Eye irritation/discomfort	238	22.9
Cough lasting >2 weeks	208	20.0
Asthmatic attack	31	3.0
